# Disentangling white-matter damage from physiological fibre orientation dispersion in multiple sclerosis

**DOI:** 10.1093/braincomms/fcaa077

**Published:** 2020-06-08

**Authors:** Kasper Winther Andersen, Samo Lasič, Henrik Lundell, Markus Nilsson, Daniel Topgaard, Finn Sellebjerg, Filip Szczepankiewicz, Hartwig Roman Siebner, Morten Blinkenberg, Tim B Dyrby

**Affiliations:** f1 Danish Research Centre for Magnetic Resonance, Centre for Functional and Diagnostic Imaging and Research, Copenhagen University Hospital Hvidovre, 2650 Hvidovre, Denmark; f2 Random Walk Imaging, AB, 222 24 Lund, Sweden; f3 Department of Radiology, Clinical Sciences, Lund, Lund University, 221 00 Lund, Sweden; f4 Division of Physical Chemistry, Department of Chemistry, Lund University, 221 00 Lund, Sweden; f5 Danish Multiple Sclerosis Center, Copenhagen University Hospital, Rigshospitalet, 2100 Copenhagen, Denmark; f6 Department of Medical Radiation Physics, Clinical Sciences, Lund, Lund University, 221 00 Lund, Sweden; f7 Department of Clinical Medicine, Faculty of Health and Medical Sciences, University of Copenhagen, 2200 Copenhagen N, Denmark; f8 Department of Neurology, Copenhagen University Hospital Bispebjerg, 2400 Copenhagen NV, Denmark; f9 Department of Applied Mathematics and Computer Science, Technical University of Denmark, 2700 Kongens Lyngby, Denmark

**Keywords:** multiple sclerosis, microscopic fractional anisotropy, diffusion MRI, fibre orientation dispersion, tensor-valued diffusion encoding

## Abstract

Multiple sclerosis leads to diffuse damage of the central nervous system, affecting also the normal-appearing white matter. Demyelination and axonal degeneration reduce regional fractional anisotropy in normal-appearing white matter, which can be routinely mapped with diffusion tensor imaging. However, the standard fractional anisotropy metric is also sensitive to physiological variations in orientation dispersion of white matter fibres. This complicates the detection of disease-related damage in large parts of cerebral white matter where microstructure physiologically displays a high degree of fibre dispersion. To resolve this ambiguity, we employed a novel tensor-valued encoding method for diffusion MRI, which yields a microscopic fractional anisotropy metric that is unaffected by regional variations in orientation dispersion. In 26 patients with relapsing-remitting multiple sclerosis, 14 patients with primary-progressive multiple sclerosis and 27 age-matched healthy controls, we compared standard fractional anisotropy mapping with the novel microscopic fractional anisotropy mapping method, focusing on normal-appearing white matter. Mean microscopic fractional anisotropy and standard fractional anisotropy of normal-appearing white matter were significantly reduced in both patient groups relative to healthy controls, but microscopic fractional anisotropy yielded a better reflection of disease-related white-matter alterations. The reduction in mean microscopic fractional anisotropy showed a significant positive linear relationship with physical disability, as reflected by the expanded disability status scale. Mean reduction of microscopic fractional anisotropy in normal-appearing white matter also scaled positively with individual cognitive dysfunction, as measured with the symbol digit modality test. Mean microscopic fractional anisotropy reduction in normal-appearing white matter also showed a positive relationship with total white-matter lesion load as well as lesion load in specific tract systems. None of these relationships between normal-appearing white-matter microstructure and clinical, cognitive or structural measures emerged when using mean fractional anisotropy. Together, the results provide converging evidence that microscopic fractional anisotropy mapping substantially advances the assessment of cerebral white matter in multiple sclerosis by disentangling microstructure damage from variations in physiological fibre orientation dispersion at the stage of data acquisition. Since tensor-valued encoding can be implemented in routine diffusion MRI, microscopic fractional anisotropy mapping bears considerable potential for the future assessment of disease progression in normal-appearing white matter in both relapsing-remitting and progressive forms of multiple sclerosis as well as other white-matter-related brain diseases.

## Teaser

Microscopic fractional anisotropy is a novel MRI technique which overcomes a fundamental limitation with the well-known crossing-fibre problem in fractional anisotropy. Andersen *et al.* showed that microscopic fractional anisotropy, much better than fractional anisotropy, reveals group differences and relates to cognitive and clinical scores in patients with multiple sclerosis.

## Graphical Abstract

**Graphical Abstract:**
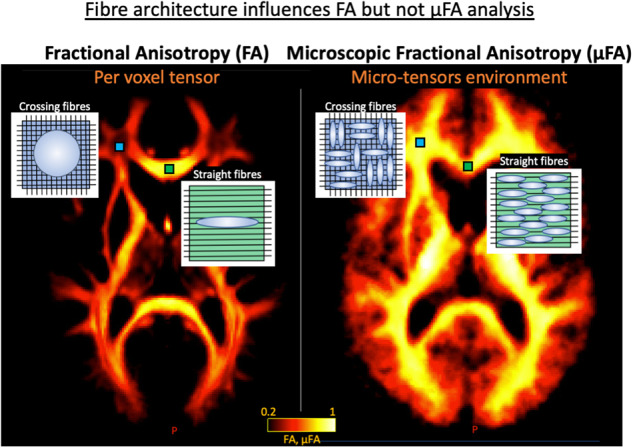

## Introduction

Multiple sclerosis is a heterogeneous neurodegenerative and inflammatory disease, causing wide-spread pathology in the central nervous system (Thompson *et al.*, 2018). MRI plays a critical role in the clinical assessment of multiple sclerosis revealing spatial dissemination and load (Filippi *et al.*, 2019). Lesion load correlates with clinical scores and disease progression (Bodini *et al.*, 2011; Matias-Guiu *et al.*, 2018). However, correlation is often rather poor and routine MRI lacks specificity to the underlying pathological mechanisms of multiple sclerosis (Poloni *et al.*, 2011). Multiple sclerosis lesions also give rise to wide-spread changes in the surrounding normal-appearing white matter (NAWM) such as axonal degeneration and demyelination, however, these changes are poorly visualized and quantified by standard diagnostic MRI techniques (Lassmann, 2011; Filippi *et al.*, 2012; van Horssen *et al.*, 2012; Kutzelnigg and Lassmann, 2014). These limitations explain why the pathological changes, as revealed by clinical MRI, only show a relatively poor correlation with individual clinical disability, contributing to the so-called clinico-radiological paradox (Chard and Trip, 2017).

Diffusion MRI measures the regional mobility of water molecules and has the potential to offer more specific insights into the underlying microstructural changes (Alexander *et al.*, 2019; Dyrby *et al.*, 2018). The diffusion of water molecules is higher along than perpendicular to axons, causing a high degree of diffusion anisotropy in cerebral white matter (WM). Fractional anisotropy (FA) of WM obtained from diffusion tensor imaging (DTI) (Basser and Pierpaoli, 1996) is tightly associated with the presence of the densely packed and myelinated axonal structures, but also the presence of glial cells in disease (Beaulieu, 2002). DTI in combination with tractography is highly sensitive and has enabled quantification of tract-related microstructural changes in multiple sclerosis, which correlate with lesion load (Droby *et al.*, 2015), clinical scores (Pagani *et al.*, 2005), cognitive measures (Bester *et al.*, 2013) and tract-specific physiological measures (Wahl *et al.*, 2011). However, consistent conclusions between similar studies are not guaranteed (Sbardella *et al.*, 2013). For example, DTI group differences between healthy controls and relapsing-remitting multiple sclerosis patients have been found in some studies (Preziosa *et al.*, 2011), but not all (Nusbaum *et al.*, 2000; Wilting *et al.*, 2016).

**Figure 1:**
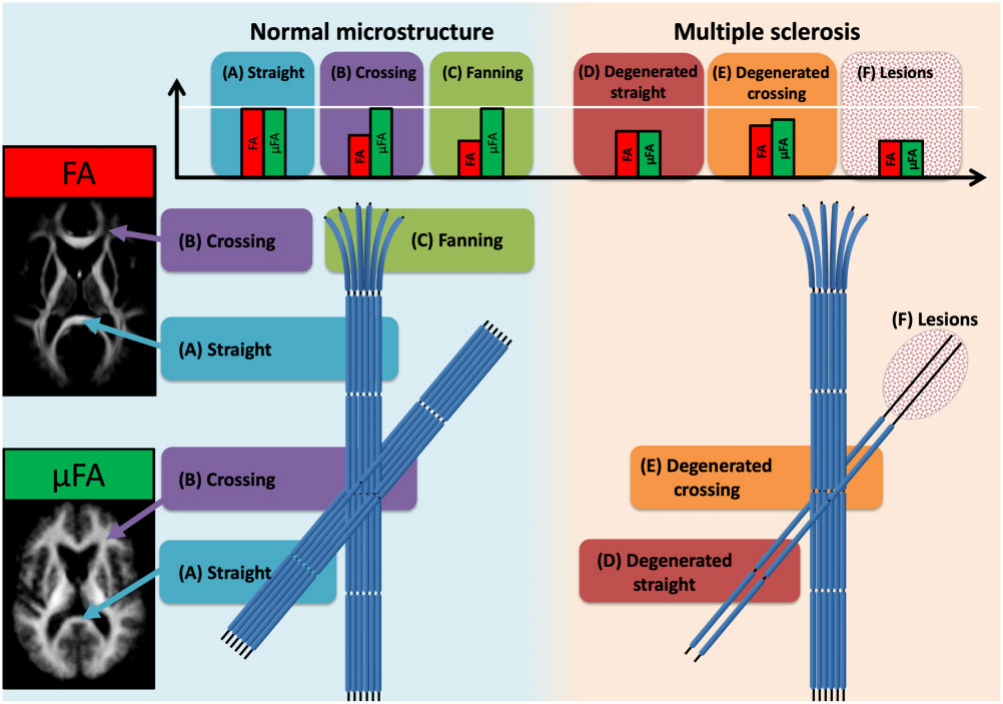
**Schematic drawing.** This schematic illustrates the expected effects on FA and μFA in various WM environments in normal tissue and damaged multiple sclerosis tissue. In addition, the figure includes voxel-wise averages of FA and μFA. In straight fibre environments (A) it is expected that both FA and μFA will be high and approximately equal, but crossing (B) and fanning (C) fibre situations will expectedly decrease FA but keep μFA high. In straight degenerated fibre bundles (D) both FA and μFA will be lower as compared with the normal tissue (A). In crossing fibres when one fibre bundle is degenerated (E) FA may be higher and μFA lower as compared with the normal tissue (B). Lesions in straight fibre bundles (F) will decrease both measures equally. FA = fractional anisotropy; μFA = microscopic fractional anisotropy.

The FA metric is challenging to interpret since it intermingles mesoscopic tissue architectonic features (i.e. fibre orientation dispersion and crossings) with microscopic tissue features (i.e. axons, cells and their density). Both can lead to changes in FA that can be interpreted as pathology as illustrated in Fig. 1. Since 90% of WM voxels contain crossing fibres (Jeurissen *et al.*, 2013; Schilling et al., 2017), axonal fibres are never perfectly aligned, making it almost impossible to disentangle tissue microstructural anisotropy from macrostructure using FA (Basser and Pierpaoli, 1996; Oouchi *et al.*, 2007; Wheeler-kingshott and Cercignani, 2009; Vos *et al.*, 2011; Wang *et al.*, 2015). For example, increased FA was reported in Alzheimer’s disease but was attributed to changes in fibre orientation dispersion rather than to changes in microscopic anisotropy (illustrated in Fig. 1, environment B versus E) (Douaud *et al.*, 2011; Teipel *et al.*, 2014).

The problems inherent to FA are introduced at the acquisition level since only a standard linear diffusion MRI (dMRI) scheme is used. However, the inherent ambiguity of FA as a microstructural measure can be circumvented by optimizing the acquisition scheme. Recently, the conventional DWI acquisition scheme has been combined with an additional tensor-valued acquisition scheme, called spherical tensor encoding (STE) (Mori and Van Zijl, 1995; Eriksson *et al.*, 2015; Westin *et al.*, 2016; Topgaard, 2017). The combination of the two dMRI acquisitions allows the estimation of a micro-FA (μFA) map which decouples fibre orientation dispersion from microstructural tissue features without the need of advanced modelling (Cory *et al.*, 1990; Jespersen *et al.*, 2014; Lasič *et al.*, 2014; Shemesh *et al.*, 2016). Until now, μFA mapping has only been applied in a few case studies of multiple sclerosis (Yang *et al.*, 2018), cancer (Szczepankiewicz *et al.*, 2015, 2016; Nilsson *et al.*, 2020) and with related techniques in ageing (Lawrenz *et al.*, 2016). Therefore, it remains to be clarified whether μFA can advance microstructural mapping of disease-related tissue damage in multiple sclerosis disease progression and whether the μFA metric correlates more closely with clinical scores than the standard FA metric.

In this study, we employed μFA mapping to resolve the ambiguity of microstructural anisotropy mapping in multiple sclerosis by disentangling disease-related tissue damage from fibre orientation dispersion. We hypothesized that μFA mapping will improve the sensitivity of diffusion MRI to detect regional microstructural tissue pathology and improve the correlation of WM tissue damage with clinical scores in individual patients relative to standard FA mapping. We applied standard dMRI (DTI) for FA mapping and tensor-valued dMRI for μFA mapping (Lasič *et al.*, 2014) in three subject groups including relapsing-remitting and primary-progressive multiple sclerosis and age-matched healthy controls. Both FA and the novel μFA were evaluated in regions of interests (ROIs) including the total NAWM as well as in three fibre tracts with different known functional relations to multiple sclerosis, and complexity in structural architecture that the μFA is expected insensitive to but not the FA. These are the corticospinal tract (CST), superior longitudinal fasciculus (SLF), and cingulum (CING). In addition, we performed correlations with clinical disability as measured with the expanded disability status scale (EDSS), cognitive processing as measured with the symbol digit modality test (SDMT).

## Materials and methods

### Subjects

The study included 45 multiple sclerosis patients and 28 healthy controls (Table 1). Five multiple sclerosis patients and one healthy control were excluded from data analysis because of missing data (data were not exported correctly from the scanner), thus the final analyses included data from 40 multiple sclerosis patients (26 relapsing-remitting multiple sclerosis, 14 primary-progressive multiple sclerosis) and 27 healthy controls. Approval was given by the Ethics committee of the Capital Region of Denmark (Protocol H-15006964) and written informed consent was obtained from all subjects before inclusion in the study according to the Declaration of Helsinki.

**Table 1:** Subject demographics

	Healthy controls	Multiple sclerosis patients	Relapsing-remitting multiple sclerosis	Primary-progressive multiple sclerosis
Number of subjects	27	40	26	14
Median (std) age	43.5 (12.0)	46.3 (11.5)	40.2 (8.9)	57.7 (5.0)
Mean (std) SDMT	50.5 (7.5)	53.3 (11.8)	57.2 (11.0)	45.9 (9.8)
Median (range) EDSS	Na	3 (0-7)	2 (0-7)	4 (3-6.5)
Median (std) lesion load (ml)	Na	10.4 (13.0)	6.5 (7.8)	17.6 (17.4)
Median (range) lesion number	Na	31 (2–108)	31 (2–97)	34 (14–108)

### Clinical and cognitive assessments

The EDSS (Kurtzke, 1983) was performed by a trained neurologist and used to examine the multiple sclerosis patients’ physical disability. The SDMT (Smith, 1982) was performed to evaluate cognitive processing speed. The multiple sclerosis patients performed the SDMT test orally, where the patients had the paper in front of them and spoke out the answer to the examiner, who noted the answer. The healthy controls did a written version of the test and wrote down the answers themselves.

### Magnetic resonance imaging

All MR images were acquired on a Philips Achieva 3T scanner using a 32-channel head-coil. Three different protocols were acquired: (i) standard clinical structural MRI for lesion drawing and segmentation of the brain’s WM; (ii) multi-shell dMRI for FA and tractography; and (iii) tensor-valued dMRI for μFA estimation. The protocols were a subset of a more comprehensive multiple sclerosis study.

*Clinical structural MRI* included T_1_-weighted [repetition time (TR) = 6.03 ms, echo time (TE) = 2.70 ms, field-of-view (FOV) = (208.25, 245, 245) mm, and with 0.85 mm isotropic voxel resolution], T_2_-weighted [TR = 2500 ms, TE = 271 ms, FOV = (190, 245, 245) mm, 0.85 mm isotropic voxel resolution] and FLAIR [TR = 4800 ms, TE = 330 ms, FOV = (202, 256, 256) mm, 1 mm isotropic voxel resolution].

*Multi-shell dMRI* was acquired using three different *b*-values with a pulsed gradient spin echo (i.e. LTE) echo-planar imaging sequence: *b*-values = (300, 1000, 2000) s/mm^2^ in (6, 50, 50) non-collinear directions evenly distributed on the unit sphere (scan time around 21.5 min). In addition, three *b* = 0 s/mm^2^ volumes were acquired with both similar and reversed phase encoding for image distortion correction (see below). The imaging parameters were: TR = 12 200 ms, TE = 70 ms, 2 mm isotropic voxel resolution, no gap between slices, 50 axial slices, FOV = (224, 224, 100) mm. The FOV covered the whole brain.

**Figure 2:**
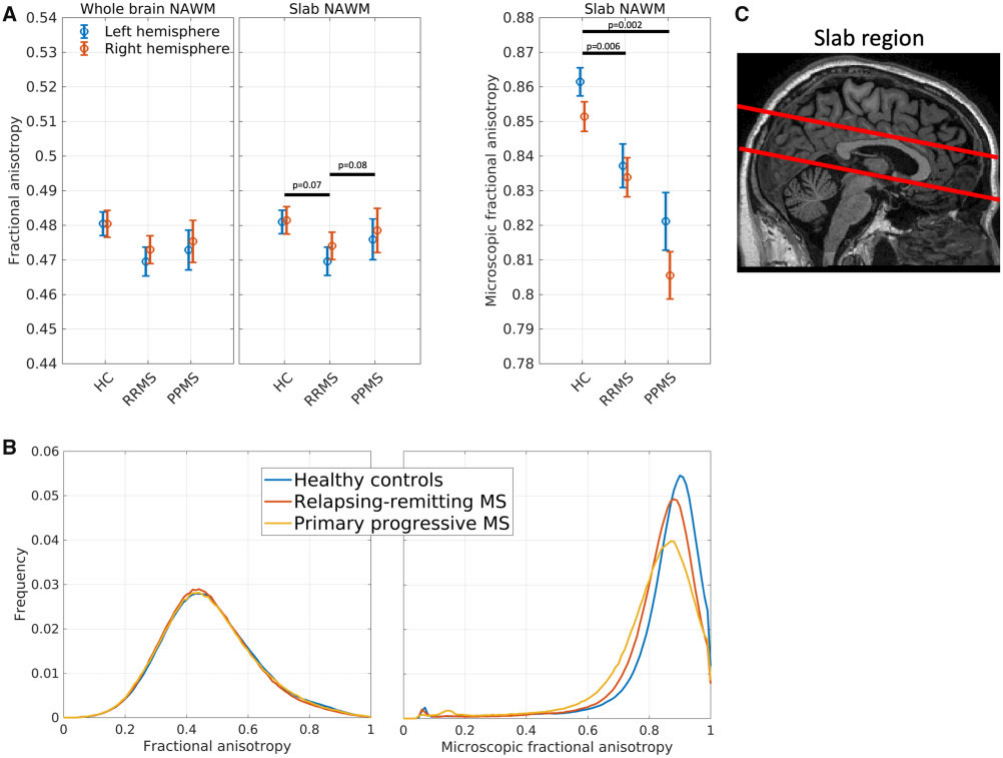
**Group average fractional anisotropy and microscopic anisotropy.** (**A**) Left: group FA averages within the whole-brain NAWM; middle: FA group averages within a slab of NAWM; right: μFA group averages within a slab of NAWM. Error bars indicate standard error of the mean. (**B**) FA (left panel) and μFA (right panel) histograms of NAWM. The histograms were estimated within each subject and then averaged within groups. (**C**) Imaging slap where microscopic fractional anisotropy was acquired. RRMS = relapsing-remitting multiple sclerosis; PPMS = primary-progressive multiple sclerosis.

*Tensor-valued dMRI* was acquired using two tensor shapes of diffusion encodings i.e. LTE and STE for six *b*-values (100, 500, 900, 1200, 1600 and 2000 s/mm^2^). The LTE acquisition was performed in 15 non-collinear directions for each shell distributed on the unit spheres as described in Szczepankiewicz *et al.* (2019) (scan time around 16.2 min). The STE uses an isotropic diffusion encoding (Mori and Van Zijl, 1995) based on the magic angle spinning of the q-vector (qMAS) (Eriksson *et al.*, 2013; Topgaard, 2013), and was acquired in 15 repetitions. Spin-echo-based echo-planar imaging was used, with TR = 5400 ms, TE = 147 ms, 2.5 mm isotropic resolution, slice gap = 0.1 mm, 14 axial slices, 90 images, FOV = (240, 240, 36.3) mm^3^. The FOV (shown in Fig. 2A) covered the axial plans of corpus callosum where lesions typically are to be found. Here we used the Philips scanner implementation of the tensor-valued dMRI sequences that also are available for other scanner platforms as described here: https://github.com/filip-szczepankiewicz/fwf_seq_resources.

### Structural MRI processing

In multiple sclerosis patients, lesions were segmented using a semi-automated method in native space. This included co-registration of the T_2_-weighted and FLAIR images to the T_1_-weighted image followed by rigid-body co-registration to the Montreal Neurological Institute (MNI) template using SPM12 software version 6470 (Welcome Department of Imaging Neuroscience, London, UK). Note that no warping was involved during this step but only rigid-body translation/rotations to align the brains best possible to standard space. Then lesions were segmented using a local thresholding technique based on the FLAIR image (Jim 6.0 Xinapse System, Leicester, UK) by a single experienced observer blinded to the subject identity. The T_1_-weighted and T_2_-weighted images were used to guide the lesion delineation. For each subject, the total number of lesions and their volume was calculated. Healthy controls also followed the first initial co-registration between T_1_-weighted, T_2_-weighted and FLAIR images. However, WM abnormalities were not manually delineated in healthy controls.

Freesurfer’s *recon_all* (version 5.3.0; http://surfer.nmr.mgh.harvard.edu) (Dale *et al.*, 1999) was used for brain segmentation to obtain WM ROIs for both left and right hemispheres. Multiple sclerosis lesions, as defined by the semi-automated method described above, were excluded from the WM to provide ROIs for NAWM. ROIs were then eroded by 2 voxels to reduce partial volume effects from CSF, GM and lesions. These ROIs were used as WM ROIs for healthy controls, and as NAWM ROIs for multiple sclerosis patients.

### Multi-shell dMRI dataset processing and FA estimation

Data were corrected for susceptibility artefacts using FSL’s *topup* based on the sets of *b* = 0 images with opposite phase encoding (Andersson *et al.*, 2003) and corrected for motion and eddy current artefacts using FSL’s *eddy* (Andersson and Sotiropoulos, 2016). This was followed by linear co-registration to the subject’s T_1_-weighted scan including re-slicing to 1 mm isotropic resolution using FSL’s *FLIRT* with sinc interpolation (Dyrby *et al.*, 2014).

Voxel-wise FA maps we estimated based on co-registered and interpolated dMRI data using MRtrix3’s *dwi2tensor* (http://www.mrtrix.org/). For the FA parameter estimation, only the *b* = 1000 s/mm^2^ and *b* = 0 s/mm^2^ images from the multi-shell dMRI protocol were used.

### Tensor-valued dMRI data processing and μFA estimation

The tensor-valued data were corrected for motion and eddy-currents in *ElastiX* (Klein *et al.*, 2010) using extrapolated reference images (Nilsson *et al.*, 2015). After correction, a mild smoothing (1.25 mm FWHM) was applied using a Gaussian kernel. The μFA map was then estimated using the Multidimensional diffusion MRI toolbox, available at https://github.com/markus-nilsson/md-dmri (Nilsson *et al.*, 2018). First, the LTE data were powder (directionality) averaged for each *b*-value, and the repeated STE dataset was averaged to gain signal-to-noise ratio. Laplace transform of gamma distribution was first fitted to the data points of LTE and STE using non-negative least-squares fitting and voxel-wise μFA maps were estimated (Lasič *et al.*, 2014). After fitting, linear co-registration to the subject’s T_1_-weighted space was performed by first co-registering the *b* = 100 s/mm^2^ image to the T_1_-weighted registered *b* = 0 s/mm^2^ image from the multi-shell dataset and then applying the same transformation to the μFA image including re-slicing to 1 mm isotropic resolution using FSL’s *FLIRT* with sinc interpolation.

Since μFA data were only acquired in a 36.4 mm slab covering the corpus callosum, we averaged FA both in the same slab as well as in the whole NAWM. The FA results are consistent for both the whole brain as well as the slab, so we only report statistics for the slab NAWM but visualize the results for both analyses.

### Tract-based segmentation using tractography

Constrained spherical deconvolution using all *b*-values from the whole brain multi-shell dataset was used to estimate individual subjects’ fibre orientation distribution images using *MRtrix3’s dwi2response* (*dhollander* method) and *dwi2fod*. A group fibre orientation distribution template from 65 subjects (including both multiple sclerosis patients and healthy controls) was generated using *MRtrix3’s population_template.* In this group template space, whole-brain probabilistic tractography was performed using default parameters (step size: 0.5 mm, max angle: 45 degrees, fibre orientation distribution-threshold = 0.1) and 10 million streamlines to estimate consistent tract systems of the brain. *Post hoc*, streamlines belonging to either SLF, CST or CING in each hemisphere were extracted by filtering the set of all streamlines. These tracts have shown to be affected and show relation to cognitive and clinical scores in multiple sclerosis patients (Liu *et al.*, 2012; Onu *et al.*, 2012; Koenig *et al.*, 2015; Tovar-Moll *et al.*, 2015; Deppe *et al.*, 2016). For each tract, this was done by manually placing four different inclusion ROIs along the tract. Finally, the number of streamlines passing through each imaging voxel was counted and thresholded to obtain a binary mask of each tract (see Supplementary Fig. 1). The thresholds used were 50 streamlines for SLF and CING and 500 for CST. The binary masks were then warped back into each individual subjects’ T_1_-weighted space. In multiple sclerosis patients, individual subject lesions overlaying any of the masks were excluded to only include NAWM with each tract. Finally, the masks were eroded by 1 voxel to reduce partial volume effects.

### Statistical analysis

Mean FA and μFA were calculated in each subject’s NAWM, CST, SLF and CING masks for both hemispheres separately. Repeated measures ‘hemisphere’ (left, right) by ‘group’ (RRMS, PPMS, healthy controls) ANOVA was performed in SPSS version 25 with age and gender as covariates. This analysis was performed separately for the total WM and NAWM ROI as well as for the CST, SLF and CING. Analysis of the linear correlation (Pearson) for FA and μFA with age, SDMT and EDSS were performed in MATLAB. Total lesion load (volume) was correlated with FA and μFA in the specific ROIs using Spearman’s rank correlations in MATLAB. In addition, lesion load was correlated with FA and μFA within ROIs. We set the significance level to *P* < 0.05 and follow-up *t*-tests in the ANOVA analyses were corrected for multiple comparisons using Bonferroni correction. SPSS provides Bonferroni corrected *P*-values (*P*_bonf_) by multiplying the un-corrected *P*-values with the number of comparisons performed, so the same significance level (*P*_bonf_ < 0.05) still holds for the follow-up tests.

### Data availability

Fully anonymized post-processed data will be made available on reasonable request.

## Results

### Disease phenotype reflected by μFA, but not FA

Voxel-wise group average maps of FA and μFA values obtained in healthy controls are shown in Fig. 1 (left panel). These group averages illustrate the difference between mean FA and μFA in relation to WM anatomy: FA values are low in regions with orientationally dispersed fibre populations, whereas μFA values are relatively homogeneous in the whole WM. Regional FA is lower than regional μFA, especially in regions with known fibres crossings, e.g. in the centrum semiovale, where corpus callosum projection and the associative fibres are crossing (Fig. 1, arrowhead B).

Repeated measures ANOVA revealed significant differences between groups, when comparing mean μFA and FA in the NAWM slab covered by dMRI, but the pattern differed for the two measures (Fig. 2). The between-group difference in μFA [*F*(2,63) = 9.00, *P* < 0.001] was driven by higher mean μFA values in the NAWM in healthy controls relative to patients with relapsing-remitting multiple sclerosis (*P*_bonf_ = 0.002) and primary-progressive multiple sclerosis (*P*_bonf_ = 0.006). Mean μFA values in the NAWM did not differ significantly between the two patient groups. Mean FA values in NAWM also showed a main effect of group [*F*(2,63) = 3.63, *P* = 0.03], but follow-up *t*-tests showed only trend-wise differences in mean FA between groups. Patients with relapsing-remitting multiple sclerosis tended to have lower FA values than healthy controls (*P*_bonf_ = 0.07) and patients with primary-progressive multiple sclerosis (*P*_bonf_ = 0.08). In contrast to mean μFA values in the NAWM, mean FA values of patients with primary-progressive multiple sclerosis did not differ from mean FA values of healthy controls. For mean μFA and FA of NAWM, ANOVA revealed no main effect of hemisphere nor group by hemisphere interaction (Fig. 2A).

### Thresholding FA impacts disease-related information

In the analysis above, we averaged FA and μFA values across all voxels in NAWM. The left panel in Fig. 2B shows the within-group average histograms of both FA and μFA measures across the whole bilateral NAWM mask. The FA histograms for the three groups overlap each other almost perfectly. However, the histograms for μFA are clearly different between the groups with primary-progressive multiple sclerosis showing a larger tail towards smaller μFA values and a lower and more left-skewed peak compared to the relapsing-remitting multiple sclerosis distribution.

A common approach to overcome the crossing-fibre problem in FA analyses is to limit the regions of interest to only include voxels with FA greater than a given threshold. Therefore, we investigated the effect of removing voxels with low FA values before calculating the mean μFA and FA values in the WM. When discarding voxels from the analysis, where FA is, respectively, lower than 0.2, 0.4 and 0.6, the between-group differences in mean FA remained the same, but group means are shifted towards higher values (Supplementary Fig. 2, left panel). The right panel in Supplementary Fig. 2 shows group means for μFA values, again, when removing voxels with low FA values. In this analysis, the group difference between the two multiple sclerosis groups was diminished when voxels with FA values <0.4 were removed, suggesting that this approach of restricting the ROI is removing microstructure information for primary-progressive patients only.

### Correlation between μFA measures and clinical rating scales

**Figure 3:**
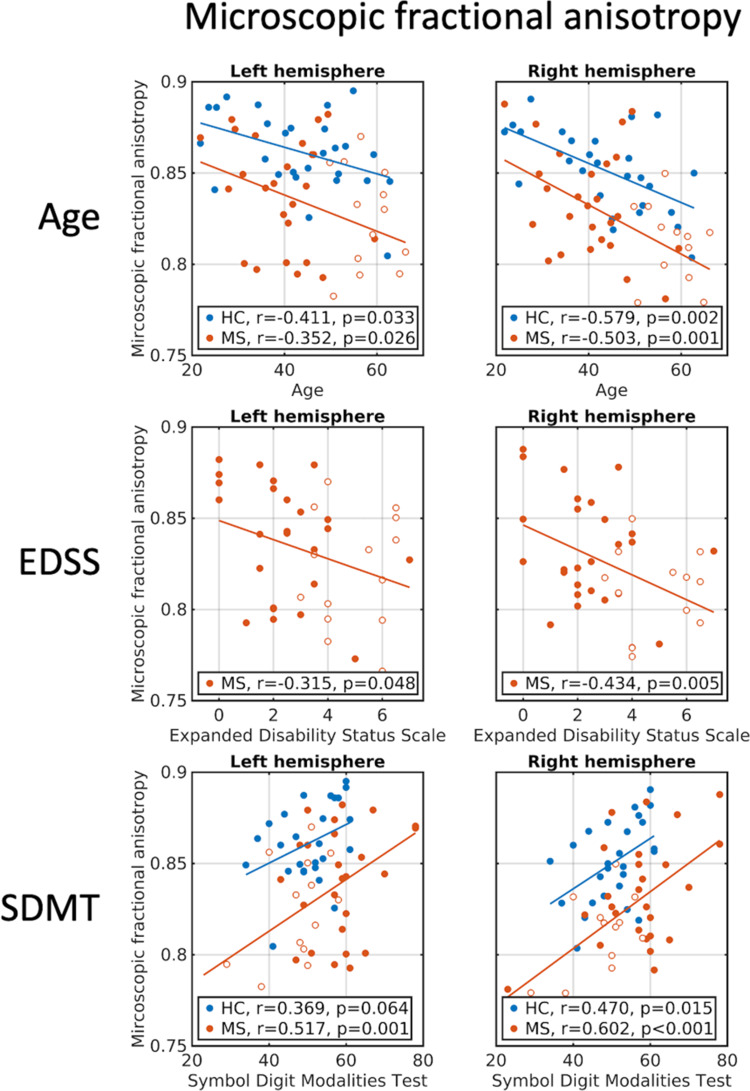
**Correlations between μFA and clinical/cognitive scores.** Age, EDSS and SDMT correlations with μFA averaged within left and right hemispheres of NAWM. First row: correlations with age. Second row: correlations with EDSS. Third row: correlations with SDMT. Blue circles: healthy controls. Full red circles: relapsing-remitting multiple sclerosis patients. Open red circles: primary-progressive multiple sclerosis patients. EDSS = expanded disability status scale; SDMT = symbol digit modalities test.

**Figure 4:**
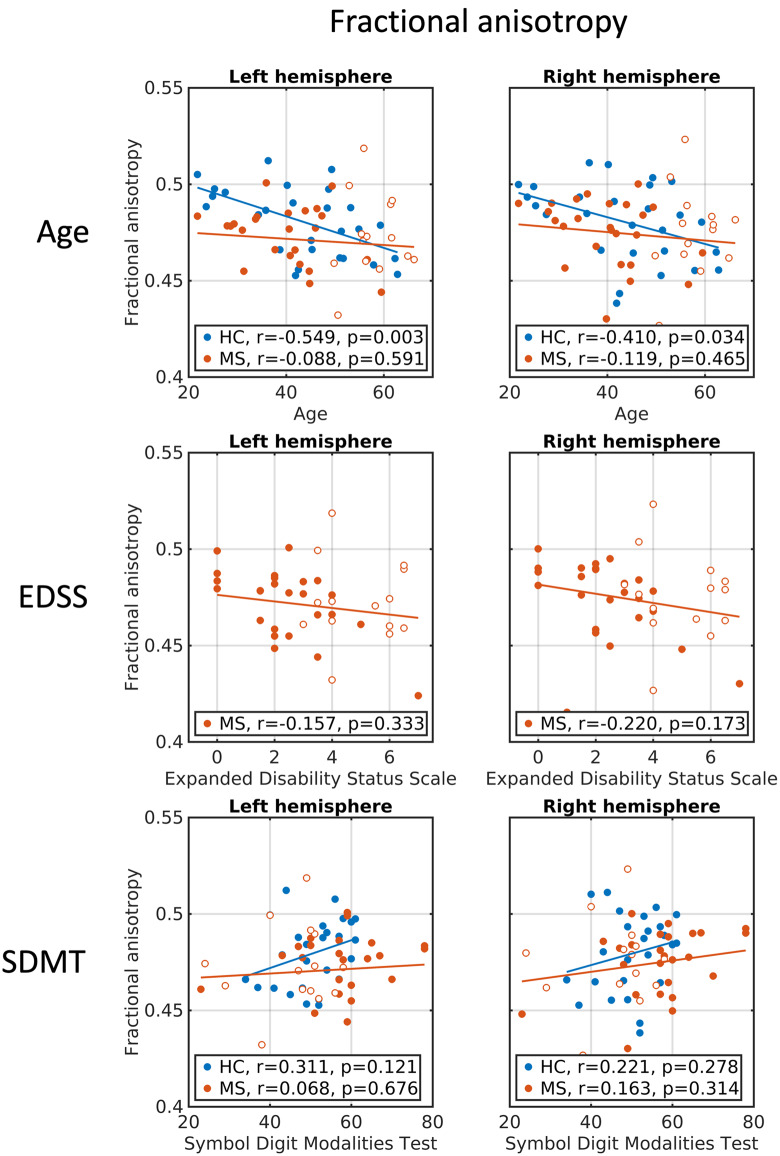
**Correlations between FA and clinical/cognitive scores.** Age, EDSS and SDMT correlations with FA (slab) averaged within left and right hemispheres of NAWM. First row: correlations with age. Second row: correlations with EDSS. Third row: correlations with SDMT. Blue circles: healthy controls. Full red circles: relapsing-remitting multiple sclerosis patients. Open red circles: primary-progressive multiple sclerosis patients. EDSS = expanded disability status scale; SDMT = symbol digit modalities test.

In the combined multiple sclerosis group (relapsing-remitting and primary-progressive multiple sclerosis) as well as in the group of healthy controls, μFA averaged in NAWM correlated negatively with age (first row in Fig. 3 and Supplementary Table 1), whereas for FA, the age relationship was only significant in the healthy control group (first row in Fig. 4 and Supplementary Table 1). However, *post hoc* tests revealed no interactions between group and age for μFA and FA. In the combined multiple sclerosis group, μFA, but not FA, was negatively correlated with physical disability as measured with the EDSS in both hemispheres. This means that lower μFA values were associated with higher disability (second row in Figs 3 and 4 and Supplementary Table 1). The correlation, however, was not significant when adjusting for age, suggesting a relation between age and EDSS. Additionally, μFA values showed significant correlations with cognitive performance as measured with the SDMT in both healthy controls and in multiple sclerosis (third row in Fig. 3), which remained significant when controlling for age in the multiple sclerosis group. Again, no significant correlations between FA and cognitive performance were found (third row in Fig. 4).

### Tract-specific group analyses

To investigate how fibre crossings along specific tracts might affect group results of FA mapping but not μFA, we performed the same set of analyses which we had performed for the entire NAWM for the NAWM belonging to specific WM tracts having different degrees of architecture complexity. The CING bundle on the part superior to mid-sagittal corpus callosum fibres was selected, because it contains (in that region) a limited number of crossing fibres. In addition, we selected the SLF and CST, both having trajectories projecting through regions where other fibre bundles are crossing e.g. with the corpus callosum (Schmahmann and Pandya, 2006).

We found no significant differences in regional μFA values among the three groups in any of the three tracts (right column in Supplementary Fig. 3 and Table 2). Yet there was a trend towards a between-group difference for μFA values in the SLF (*P* = 0.085) as well as a trend towards an effect of hemisphere in the CST (*P* = 0.062). Interestingly, albeit not being significant, regional μFA values showed a consistent similar pattern in all tract systems with a relative decrease in μFA values from healthy controls to relapsing-remitting to primary-progressive multiple sclerosis. In contrast, regional FA values showed significant group differences in the CST (main effect of group: *P* = 0.007). This between-group difference was caused by higher FA values in patients with primary-progssive multiple sclerosis relative to patients with relapsing-remitting multiple sclerosis (*P*_bonf_ = 0.024) and healthy controls (*P*_bonf_ = 0.006). Increase in FA with disease progression could indicate degeneration of a crossing-fibre tract (Fig. 1E). When considering all patients with multiple sclerosis together, we found negative correlations between age and μFA values in SLF (both hemispheres) and left CING (Supplementary Table 1). Similarly, μFA values in left CING of healthy controls also correlated with age. In the multiple sclerosis group, disability as measured with EDSS correlated negatively with μFA values in the left CING. Trends towards significant correlations were also found in left CST and right SLF. However, when controlling for age these effects were no longer significant. FA values in the left SLF (whole brain) correlated *positively* with EDSS, which also persisted after controlling for age.

Both, tract-specific μFA and FA values, scaled with cognitive processing speed in patients with multiple sclerosis, as measured with SDMT. SDMT correlated positively with tract-specific μFA values in bilateral CING and SLF. Trends towards significant correlation between tract-specific μFA values and cognitive processing speed were also found in the right CST. In healthy controls, significant correlations between tract-specific μFA values and cognitive processing speed were observed in the right CING and right SLF. There, was also a significant relationship between cognitive processing speed and tract-specific FA values, but this correlation was only found for the left CING and was present in both, multiple sclerosis and healthy controls.

### Whole-brain lesions-load correlates with mean μFA but not FA of NAWM

The hallmark of multiple sclerosis is the focal lesions, which are distributed diffusely in the brain. Lesions are areas with a high degree of demyelination and axonal degeneration and will thus also affect the remaining part of the NAWM. We correlated lesion load, that is the total lesion volume, with μFA values in the different regions of interest to investigate whether a larger number of lesions would result in greater decrease of microstructural anisotropy.

Whole-brain lesion load correlated with mean μFA of NAWM in the right and left hemisphere (left *r* = −0.41, *P* = 0.009, right *r* = −0.56, *P* < 0.001) as well as all of the individual ROIs (left CING: *r* = −0.37, *P* = 0.02; right CING: *r* = −0.46, *P* = 0.003; left CST: *r* = −0.38, *P* = 0.02; right CST: *r* = −0.44, *P* = 0.005; left SLF: *r* = −0.53, *P* = 0.001; right SLF: *r* = −0.53, *P* < 0.001, all Spearman’s rank correlations). This showed that higher extent of global lesion load was associated with lower mean μFA in the NAWM. This was also true when correlating μFA values with lesion load within individual tracts of NAWM, see Supplementary Fig. 4. Only in the right SLF (*r* = −0.32, *P* = 0.048) and left SLF (trend *r* = −0.27, *P* = 0.09) correlated FA with whole-brain lesion load. We did not find a direct relation between lesion load and EDSS (*r* = 0.13, *P* = 0.41) nor SDMT (*r* = −0.25, *P* = 0.12).

## Discussion

We show that μFA mapping substantially advances microstructural imaging of cerebral WM with multiple sclerosis compared to standard DTI-based FA mapping. Tensor-valued dMRI minimized the influence of fibre dispersion to the estimation of regional anisotropy already at the data acquisition stage (Lasič *et al.*, 2014). This rendered diffusion-based microstructural mapping more sensitive to microstructural alterations directly caused by pathology and overcome the ambiguity from fibre architecture inherent to standard FA mapping. Importantly, the μFA mapping showed a consistent reduction in ‘microscopic’ anisotropy of NAWM in patients with relapsing-remitting and primary-progressive multiple sclerosis relative to the healthy control group. This significant difference between healthy individuals and patients affected by multiple sclerosis was not evident, when quantifying ‘macroscopic’ anisotropy of NAWM with DTI-based FA mapping that also is sensitivity to complex fibre architecture. Correlational analysis showed that mean μFA of NAWM scales with physical disability, cognitive dysfunction and the participant’s age. These relationships between WM microstructure and clinical variables were not evidenced by mean FA. In the correlation analyses, we pooled patients with relapsing-remitting and primary-progressive multiple sclerosis into a single patient group. We acknowledge that the two multiple sclerosis groups are two distinct phenotypes and, in our study, differed in age and EDSS distribution. Nonetheless, individual NAWM μFA values scaled linearly with patient-specific disease and clinical variables, while NAWM μFA value did not. This observation in a heterogenous patient group highlights the potential of NAWM μFA measurements to provide an MRI-based estimate of multiple sclerosis-related impairment.

Axonal degeneration and indirectly demyelination effects should reduce regional FA because of changes in the volume fractions of the cellular spaces (Beaulieu, 2002; Tuch, 2004; Wheeler-kingshott and Cercignani, 2009). Practically, our results prove that this is not necessarily the case. Most WM voxels contain crossing fibres with a considerable amount of fibre orientation dispersion (Jeurissen *et al.*, 2013; Schilling *et al.*, 2017). In these voxels, regional FA is determined by two tissue properties, namely microscopic diffusion anisotropy and fibre orientation dispersion. This renders the interpretation of regional FA values inherently ambiguous in the context of neuroinflammatory and neurodegenerative diseases. For instance, these two features may exert opposing effects on regional FA. On the one hand, axonal degeneration and demyelination effects may lower FA. On the other hand, axonal degeneration of a single fibre bundle in a crossing-fibre region may reduce the regional dispersion of fibre orientation dispersion and hereby increase regional FA (Pierpaoli *et al.*, 2001; Douaud *et al.*, 2011; Teipel *et al.*, 2014). The ambiguity of the FA metric may explain why tract-specific analysis showed higher mean FA values in patients with primary-progressive multiple sclerosis as compared with patients with relapsing-remitting multiple sclerosis and healthy controls. This effect was especially pronounced in the SLF due to its high degree of fibre orientation dispersion, underlining that regional FA is less suited to detect fibre damage in regions with large fibre orientation dispersion. Actually, the observed increasing FA value in primary-progressive multiple sclerosis could indicate a crossing-fibre environment where one fibre tract has degenerated and the fibre orientation dispersion decreases (illustration Fig. 1E), which was previously described in Alzheimer’s disease (Douaud *et al.*, 2011; Teipel *et al.*, 2014). On the other hand, regional FA and μFA in CING, a relatively homogenous fibre bundle, resembled each other due to relatively well-aligned fibres, showing a stronger relative decrease in anisotropy in patients with primary-progressive than relapsing-remitting multiple sclerosis.

We found that the total lesion load correlates negatively with μFA in the remaining NAWM as well as in different tract systems (Supplementary Fig. 2). Multiple sclerosis lesions are focal areas with demyelination and axonal degeneration, but degeneration spreads out from the lesions to the rest of the brain, affecting the microstructure of NAWM. We argue that the degree of axonal degeneration in NAWM scales with lesion load and this mechanism may account for lower mean μFA values in the NAWM of patients with high lesion load. Especially in primary-progressive multiple sclerosis, primary microscopical changes observed with histology in non-lesioned WM may also contribute to the observed μFA decrease in the NAWM (Lassmann, 2019). In our study, we did not find the same relation with FA, which only correlated negatively with global lesion load in the right SLF (as well as a trend towards negative correlation in left SLF).

Only mean μFA but not FA scaled with cognitive processing speed in patients and healthy controls. The higher the individual SDMT score, the higher was mean μFA of the NAWM. The same positive relationship was found when considering the mean μFA of the CING or SLF. Similar correlations have been described previously between SDMT and mean FA in CING (Lin *et al.*, 2014; Koenig *et al.*, 2015) and corpus callosum (Pokryszko-Dragan *et al.*, 2018). However, in our study, mean FA only correlated with SDMT in the left CING.

Mean μFA but not mean FA showed an inverse linear relationship with physical disability as measured with the EDSS score. This effect was found when using the total NAWM mask but was also present when using the NAWM in the left CING. In the existing literature, the relation between FA of NAWM and physical disability is conflicting with some studies reporting significant correlations (Liu *et al.*, 2012; Onu *et al.*, 2012; Tovar-Moll *et al.*, 2015; Deppe *et al.*, 2016) while others did not (Griffin *et al.*, 2001; Fink *et al.*, 2010; Daams *et al.*, 2016). Together, our findings suggest that μFA may better reflect disease-related functional impairment than conventional FA because it reveals changes in microstructure anisotropy independent of fibre architecture.

Both, mean μFA and mean FA correlated with age, showing lower anisotropy with increasing age in healthy controls. This finding replicates previous studies showing similar age-related effects of FA (Sullivan and Pfefferbaum, 2006) or μFA (Lawrenz *et al.*, 2016). Reduced microscopic anisotropy with age suggests a change in the volume fraction of the cellular spaces due to axonal degeneration or changes in myelination. In addition, more supporting cells, e.g. glial cells, in the microstructural environment would result in lower microscopic anisotropy (Lynch *et al.*, 2010; Al-Mashhadi *et al.*, 2015). We observed that patients with multiple sclerosis also showed lower WM anisotropy with age, but only when using μFA and not FA as anisotropy measure. The absence of a change in WM FA with patient’s age may be caused by disease-related architectural change (degeneration of a single tract in a crossing tract environment) that cause an apparent increase in FA, obscuring the age-associated reduction in anisotropy.

A common approach to overcome the crossing-fibre problem in FA analyses is to limit the regions of interest to only include voxels in which FA exceeds a given threshold (e.g. FA > 0.2). Interestingly though, our analyses showed that this did not change the relations between groups (Supplementary Fig. 2), showing consistent results across different FA-thresholds. An alternative strategy to overcome the crossing-fibre problem is to extract FA in coherent WM structures, e.g. using tract-based spatial statistics (Smith *et al*., 2006). However, both ways of limiting the analysis to voxels with coherent fibre orientation have the disadvantage of lowering the signal to noise ratio since fewer voxels are used to compute the mean statistic. Moreover, this approach excludes relevant portions of WM. Regardless of these strategies to mitigate the fibre dispersion problem, FA is modulated by effects of crossing fibres and thus cannot reflect the true underlying microscopic anisotropy due to limited spatial resolution (Budde and Frank, 2012; Schilling *et al.*, 2017). Taken together, our observations suggest that μFA better reflects the true underlying microstructural anisotropy and thus also the disease condition.

It has been proposed that μFA can be obtained using linear tensor encoding data only (Kaden *et al.*, 2016). However, this approach has been showed to be invalid (Henriques *et al.*, 2019). It has also been proposed that orientation dispersion can be accounted for by the use of biophysical models with linear tensor encoding data only (Zhang *et al.*, 2012; Kaden *et al.*, 2016), however, the result from such approaches depends as much on the assumptions in the model as on the underlying microstructure (Lampinen *et al.*, 2017, 2019; Dyrby *et al.*, 2018; Novikov *et al.*, 2019). The approach in this study did not make any assumptions on the configuration of the microstructure but instead relied on additional information provided by the STE collected at the acquisition.

Our μFA and FA results were obtained in a slab of slices covering part of the brain hence only the part of a tract projecting through the slab of slices was investigated. Focal pathological effects on the tract outside the NAWM volume covered by our slab of slices could impact our correlation results with clinical scores. However, any neuronal degeneration caused by focal attacks outside the volume covered by the NAWM slab will affect the microstructure along the entire tract also within the NAWM slab (Pierpaoli *et al.*, 2001). Therefore, tract-related NAWM changes within the slab are expected to correlate with neurodegenerative effect changes appearing along the whole tract hence to representative correlated with clinical scores.

In conclusion, μFA mapping is not affected by fibre architecture effects as conventional DTI-based FA mapping. Therefore, it is a more specific imaging tool to better capture microstructure anisotropy changes in NAWM multiple sclerosis pathology by demonstrating better group statistics in phenotype detection independent of the complexity of the tract system investigated. The μFA mapping also gives a better reflection of disease-related functional impairment when correlated with clinical scores. This motivates the future use of μFA mapping acquisitions as a complementary MRI modality to track the course of pathology dynamics and to test the efficacy of new treatments.

## Supplementary Material

fcaa077_Supplementary_Data

## Abbreviations

CSF =: cerebrospinal fluid
CING =: cingulum
CST =: corticospinal tract
dMRI =: diffusion magnetic resonance imaging
DTI =: diffusion tensor imaging
EDSS =: expanded disability status scale
FA =: fractional anisotropy
FOD =: fibre orientation distribution
LTE =: linear tensor encoding
μFA =: microscopic fractional anisotropy
MNI =: Montreal Neurological Institute
NAWM =: normal-appearing white matter
PPMS =: primary-progressive multiple sclerosis
ROI =: regions of interests
STE =: spherical tensor encoding
SLF =: superior longitudinal fasciculus
RRMS =: relapsing-remitting multiple sclerosis
SDMT =: symbol digit modality test
WM =: white matter
