# Supplementary material for: Disentangling white-matter damage from physiological fibre orientation dispersion in multiple sclerosis
Source: Brain Commun. 2020 Jun 8;2(2):fcaa077. doi: 10.1093/braincomms/fcaa077 (PMC7472898; doi:10.1093/braincomms/fcaa077)
Supplement: fcaa077_Supplementary_Data [file fcaa077_supplementary_data.pdf]

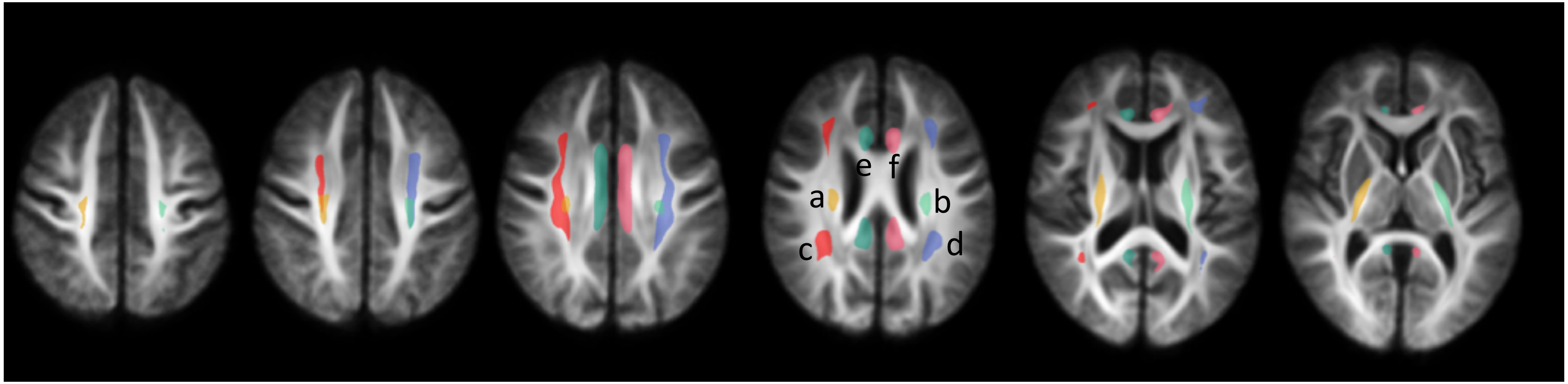

Supplementary Figure 1 | ROIs used in the study. Left (a) and right (b) corticospinal tract. Left (c) and right (d) superior longitudinal fasciculus. Left (e) and right (f) cingulum.

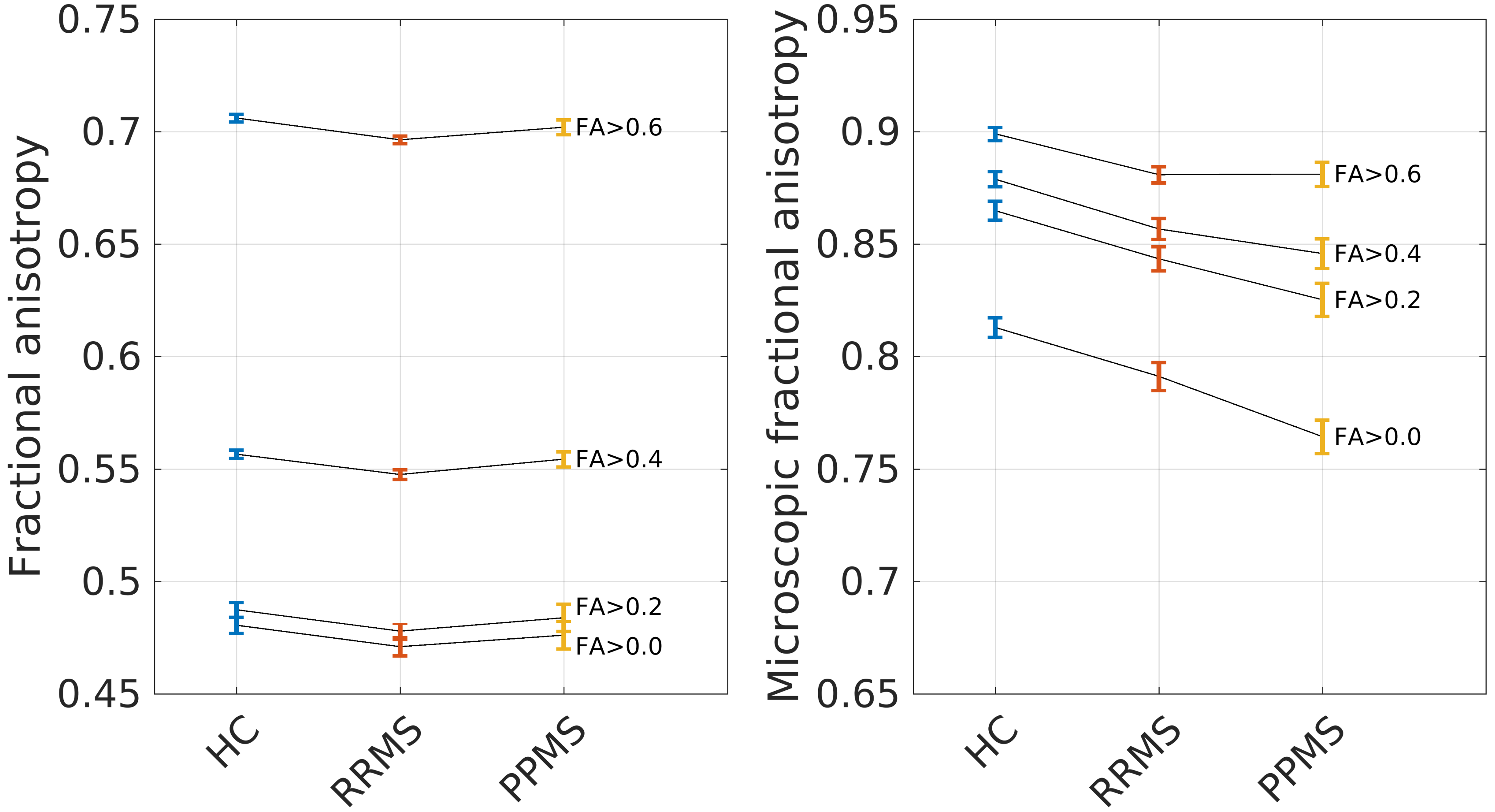

Supplementary Figure 2 | Group means (bars reflects standard error of the mean) when removing voxels with different fractional anisotropy (FA) thresholds. The thresholds used were 0.0 (i.e no threshold), 0.2, 0.4, 0.6. The relative group difference in FA remain constant for different FA thresholds. For microscopic fractional anisotropy (µFA), the relapsing-remitting multiple sclerosis and primary-progressive multiple sclerosis become more similar when discarding voxels with lower FA values from the analysis. HC = healthy controls, RRMS = relapsing-remitting multiple sclerosis, PPMS = primary progressive multiple sclerosis.

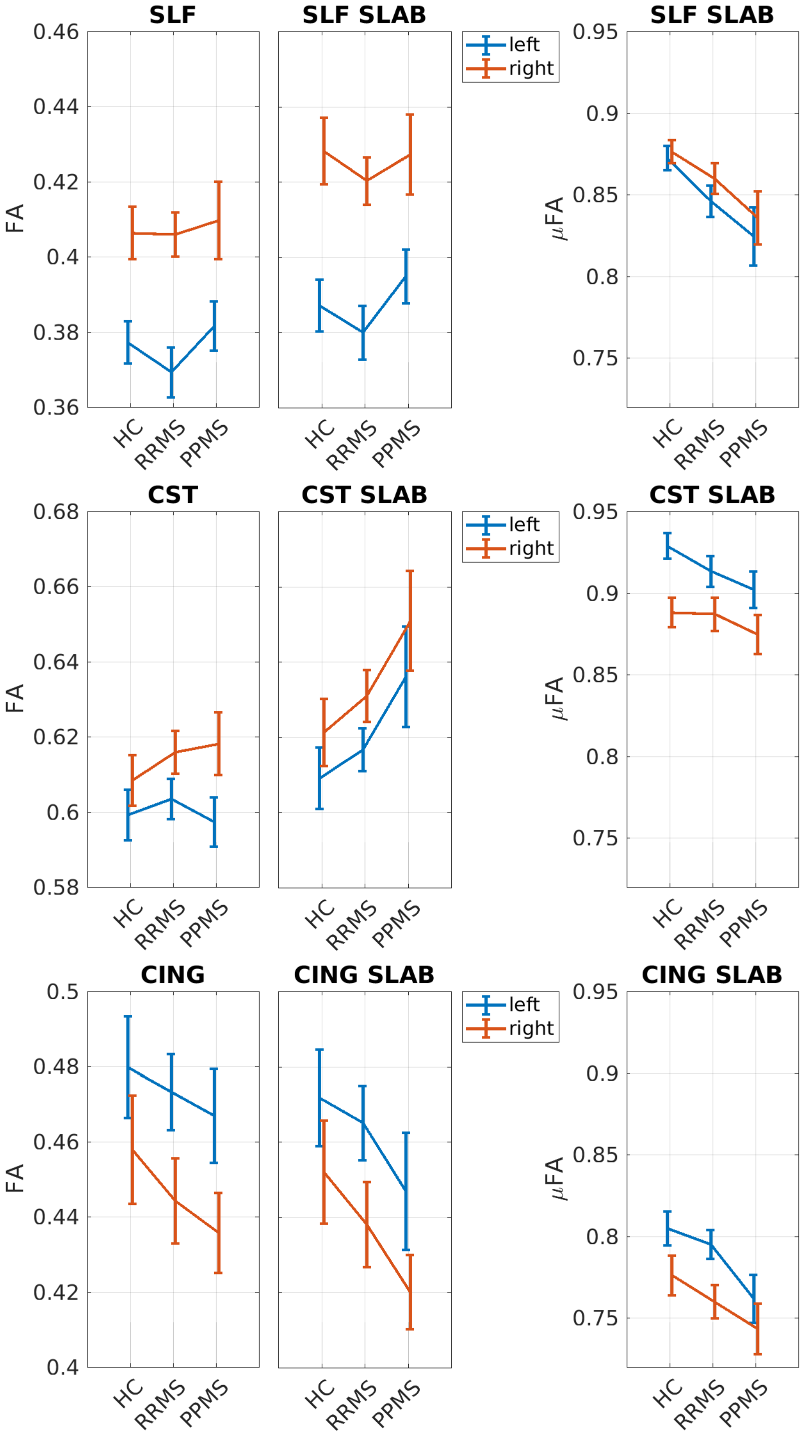

Supplementary Figure 3 | Group differences for FA and μFA. FA and μFA in superior longitudinal fasciculus (SLF) (first row), corticospinal tract (CST) (second row), cingulum (CING) (third row).
HC = healthy controls, RRMS = relapsing-remitting multiple sclerosis, PPMS = primary progressive multiple sclerosis.

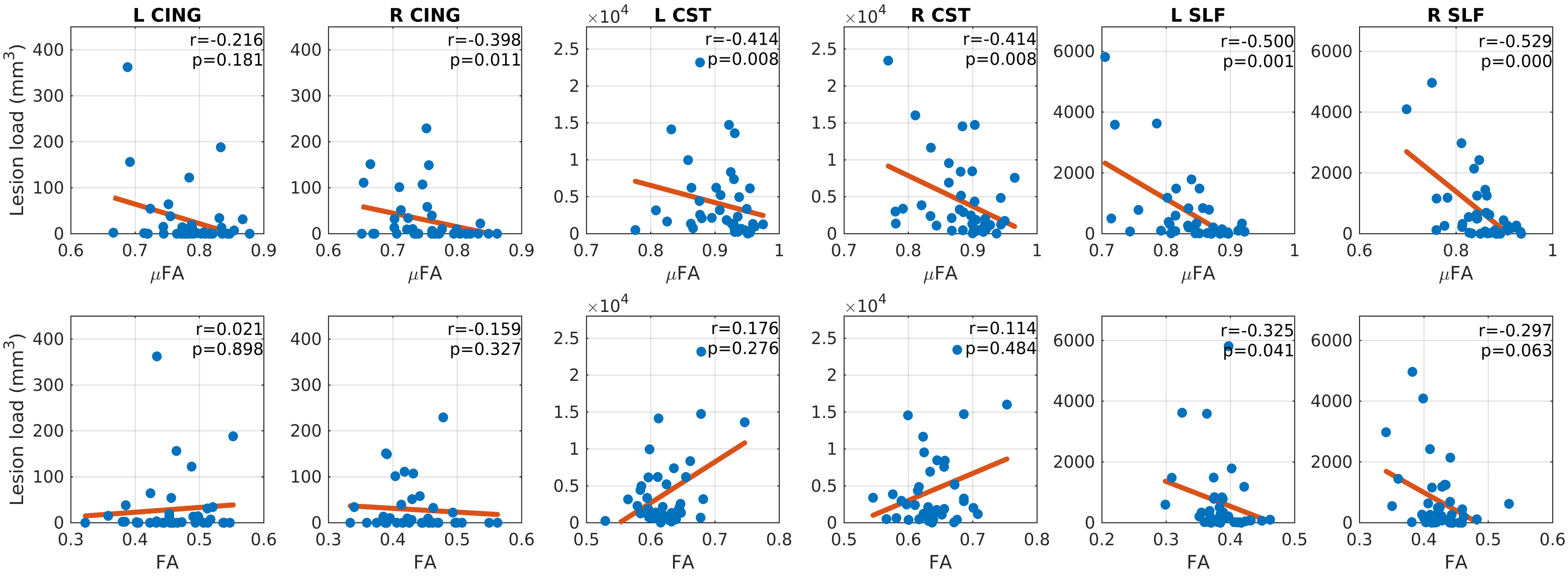

Supplementary Figure 4 | correlations between lesion load and microscopic fractional anisotropy (μFA) (first row) and fractional anisotropy (FA) (second row) in individual ROIs. L = left, R = right, CING = cingulum, CST = corticospinal tract, SLF = superior longitudinal fasciculus.

				Healthy controls				Multiple sclerosis					
				Age		SDMT		Age		SDMT		EDSS	
				r	*p*	*r*	*p*	*r*	*p*	*r*	*p*	*r*	*p*
NAWM	FA	Everything	L	**-0.588**	**0.001**	0.320	0.111	0.001	0.995	-0.010	0.953	-0.080	0.624
			R	**-0.422**	**0.028**	0.176	0.391	-0.080	0.624	0.127	0.436	-0.155	0.339
	FA	Slab	L	**-0.549**	**0.003**	0.311	0.121	-0.088	0.591	0.068	0.676	-0.157	0.333
			R	**-0.410**	**0.034**	0.221	0.278	-0.119	0.465	0.163	0.314	-0.220	0.173
	µFA		L	**-0.411**	**0.033**	*0.369*	*0.064*	**-0.352**	**0.026**	**0.517**	**0.001**	**-0.315**	**0.048**
			R	**-0.579**	**0.002**	**0.470**	**0.015**	**-0.503**	**0.001**	**0.602**	**0.000**	**-0.434**	**0.005**
CING	FA	Everything	L	**-0.393**	**0.043**	**0.469**	**0.016**	**-0.326**	**0.040**	**0.360**	**0.022**	-0.263	0.100
			R	-0.303	0.125	0.193	0.345	**-0.313**	**0.049**	0.255	0.113	-0.175	0.279
	FA	Slab	L	**-0.442**	**0.021**	**0.482**	**0.013**	-0.203	0.210	*0.274*	*0.087*	-0.161	0.321
			R	*-0.348*	*0.076*	0.206	0.314	-0.217	0.179	0.201	0.214	-0.123	0.449
	µFA		L	**-0.441**	**0.021**	0.320	0.111	**-0.378**	**0.016**	**0.549**	**0.000**	**-0.330**	**0.038**
			R	-0.238	0.233	**0.432**	**0.027**	*-0.290*	*0.069*	**0.344**	**0.030**	-0.131	0.421
CST	FA	Everything	L	**-0.482**	**0.011**	0.214	0.294	0.134	0.410	-0.225	0.163	*0.306*	*0.055*
			R	**-0.496**	**0.008**	0.161	0.433	0.146	0.368	-0.160	0.323	0.201	0.213
	FA	Slab	L	*-0.339*	*0.084*	0.147	0.473	-0.147	0.364	0.045	0.782	0.064	0.694
			R	**-0.394**	**0.042**	0.270	0.182	0.047	0.774	0.099	0.543	0.063	0.698
	µFA		L	-0.050	0.804	-0.206	0.314	-0.171	0.292	0.223	0.167	*-0.294*	*0.065*
			R	-0.032	0.876	-0.031	0.880	-0.062	0.702	*0.299*	*0.061*	-0.202	0.211
SLF	FA	Everything	L	**-0.398**	**0.040**	-0.108	0.600	0.110	0.501	-0.182	0.262	**0.364**	**0.021**
			R	*-0.339*	*0.083*	-0.188	0.358	-0.067	0.683	-0.148	0.363	0.156	0.337
	FA	Slab	L	*-0.376*	*0.053*	-0.003	0.989	0.012	0.940	-0.043	0.792	0.216	0.181
			R	-0.288	0.145	-0.100	0.625	-0.054	0.741	-0.114	0.486	0.054	0.739
	µFA		L	-0.240	0.229	0.275	0.173	**-0.360**	**0.022**	**0.527**	**0.000**	-0.233	0.148
			R	-0.235	0.238	**0.492**	**0.011**	**-0.383**	**0.015**	**0.478**	**0.002**	*-0.285*	*0.074*

Supplementary Table 1. This table shows correlations between age and SDMT within healthy controls and between age, EDSS, and SDMT in multiple sclerosis. FA = Fractional anisotropy; µFA = microscopic fractional anisotropy; EDSS = expanded disability status scale; SDMT = symbol digit modality test; L = left; R = right; CING = cingulum; CST = corticospinal tract; SLF = superior longitudinal fasciculus.

	μFA			FA		
	Group	Hemisphere	Group x hemisphere	Group	Hemisphere	Group x hemisphere
CING	F=1.07<br>p=0.35	F=2.03<br>p=0.16	F=0.19<br>p=0.83	F=0.87<br>p=0.43	F=1.16<br>p=0.29	F=0.16<br>p=0.85
CST	F=0.75<br>p=0.48	F=3.60<br>p=0.06	F=0.89<br>p=0.41	F=5.34<br>p=0.007	F=1.07<br>p=0.31	F=0.08<br>p=0.92
SLF	F=2.57<br>p=0.09	F=0.27<br>p=0.61	F=1.21<br>p=0.31	F=2.40<br>p=0.099	F=8.71<br>p=0.004	F=0.09<br>p=0.91

Supplementary Table 2 | ANOVA results within each of the tracts for FA and μFA.
CING = cingulum; CST = corticospinal tract; SLF = superior longitudinal fasciculus, μFA = microscopic fractional anisotropy, FA = fractional anisotropy.
